# Fabrication and Characterization of Intelligent Multi-Layered Biopolymer Film Incorporated with pH-Sensitive Red Cabbage Extract to Indicate Fish Freshness

**DOI:** 10.3390/polym14224914

**Published:** 2022-11-14

**Authors:** Mindu Zam, Itthi Niyumsut, Kazufumi Osako, Saroat Rawdkuen

**Affiliations:** 1 Food Science and Technology Program, School of Agro-Industry, Mae Fah Luang University, 333 Moo 1 Thasud, Chiang Rai 57100, Thailand; 2 Department of Food Science and Technology, Tokyo University of Marine Science and Technology, Tokyo 108-8477, Japan; 3 Unit of Innovative Food Packaging and Biomaterials, Mae Fah Luang University, 333 Moo 1 Thasud, Chiang Rai 57100, Thailand

**Keywords:** red cabbage extract, anthocyanin, biopolymers, biodegradable, fish freshness, multi-layer film

## Abstract

This study aimed to fabricate an intelligent monolayer and multi-layered biodegradable films incorporated with red cabbage extract (RCE) to act as a safe and reliable freshness indicator. A film-forming solution (FFS) of gelatin, carboxymethyl cellulose (CMC) and chitosan was prepared and fortified with 0.5% (*w*/*v*) of RCE for developing intelligent monolayer films. The intelligent multi-layer film was prepared via layer by layer casting of gelatin, chitosan (added with 0.5% of RCE) and CMC biopolymers. The thickness of the multi-layered film was the highest (0.123 ± 0.001 mm) compared to gelatin-, CMC- and chitosan-based monolayer films (*p* < 0.05). Chitosan film has the highest tensile strength (*p* < 0.05), followed by multi-layer, CMC and gelatin films. Elongation at break was slightly higher in CMC (35.67 ± 7.62%) compared to the multi-layer film (33.12 ± 9.88%) and gelatin film (*p* > 0.05). Water vapor permeability was higher in the multi-layer film (1.244 ± 0.05 × 10^−5^ g mm h^−1^cm^−2^ P^−1^) than the other monolayer films. Moisture content was highest in chitosan film followed by the multi-layered film (*p* < 0.05) and then the CMC and gelatin films. CMC film showed the highest solubility compared to multi-layered and chitosan film (*p* < 0.05). Additionally, transmittance and transparency values in the multi-layered film were the lowest compared to the chitosan-, CMC- and gelatin-based films. L* and a* values were the lowest, while b* values increased in the multi-layered film compared to the other film samples (*p* < 0.05). pH sensitivity and ammonia gas tests revealed similar color changes in chitosan and multi-layer films. However, FTIR spectra confirmed that dye leaching was not detected for the multi-layered film soaked in ethanol. The biodegradability test showed rapid degradation of multi-layered and chitosan films within 1 month. Based on the optimum results of the multi-layered film, it was applied to monitor the fresh quality of tilapia fish fillets at 4 °C for 10 days. The results of freshness acceptability were noted on day 6 due to the change in color of the multi-layer film with an estimated total volatile basic nitrogen content of 21.23 mg/100 g. Thus, the multi-layered film can be used as an indicator to monitor the quality of the fish freshness without leaching dye onto the food surface.

## 1. Introduction

Intelligent packaging has been widely explored to detect the safety and shelf-life of foods susceptible to microbial and chemical spoilage during storage [1]. Intelligent packaging material functions include detecting, sensing, tracking and communicating with the consumers to facilitate the decision-making processes related to the quality and safety of the product [2]. However, the majority of the proposed concepts of intelligent packaging focus on synthetic or biodegradable monolayered films. Color changes obtained from a monolayer film could happen due to contact of the film with food metabolites, moisture or dye migration [3]. Chemical indicators (such as polyaniline, methyl red and chlorophenol red) used in intelligent food packaging have potential safety problems due to migration [4]. Meanwhile intelligent films produced from synthetic polymers and synthetic dyes have raised environmental and safety concerns because of poor degradability and risks of chemical leaching that could migrate to the food products, thereby affecting the health of consumers [5].

Promising biopolymers such as gelatin, chitosan and carboxymethyl cellulose (CMC) have been employed in the preparation of intelligent biodegradable films. Gelatin is a high-molecular-weight biopolymer derived from collagen by thermal degradation [6]. Gelatin-based packaging films are non-toxic with excellent film-forming properties and mechanical properties [7]. Chitosan is an eco-friendly bio-based amino polysaccharide with potential antimicrobial, film-forming, physical and mechanical properties [8]. Moreover, CMC is a water-soluble cellulose ether, and due to its availability, biodegradability, biocompatibility, non-toxicity and good film-forming properties, it has been widely used in the preparation of bio-nanocomposite films for food processing and pharmaceutical industries [9].

Anthocyanin extracts from plant-based products have been incorporated in pH sensing intelligent films to replace chemically synthesized dyes. Anthocyanins are the natural pigments of vascular plants and are responsible for the pink, red, violet and blue colors of vegetables and fruits such as *Roselle calyx*, purple eggplant, beetroot, dragon fruit and red cabbage [10,11]. The anthocyanins in red cabbage extract are all derivatives of cyanidin (268 ± 2 μg/mg), mainly with 19% nonacylated, 51% monoacylated and 31% diacylated structures with ferulic, sinapic, *p*-coumaric and caffeic acids [12]. The stability of anthocyanin pigments is affected mostly by the pH value, being more stable at acidic mediums (2–4 pH values) [13]. Anthocyanins from red cabbage exhibit pH-specific color changes [14]. Gelatin- and gellan gum-based edible film fortified with red radish anthocyanins extract showed an orange red to yellow color change in the pH range of 2–12 to indicate milk and fish spoilage [3]. RCE contains higher content of acylated anthocyanins that show enhanced thermal and photo-stability, antioxidant capacity and a vast color spectrum in comparison with non-acylated ones [15]. Additionally, the acylation affects the chemical properties of anthocyanins as the structural size is increased, and polarity is affected based on the type of acyl group that changes the spatial organization and decreases chemical reactivity [16]. Due to the high stability and chemical properties of acylated anthocyanins, they can be considered potential sources of natural dyes.

In this study, we aimed at developing a biopolymer-based, pH-sensitive intelligent film by adding anthocyanin-rich RCE into gelatin-, chitosan- and CMC-based monolayer films and multi-layer (gelatin/chitosan + RCE/CMC) film. The RCE-anchored monolayer and multi-layer films were analyzed for microstructural, physicochemical, mechanical and disintegrable properties. Moreover, the multi-layer film was applied to monitor the freshness of tilapia fish fillets during refrigerated storage.

## 2. Materials and Methods

### 2.1. Materials

Red cabbage was procured from Makro supermarket in Chiang Rai, Thailand, in May 2022. Commercial bovine gelatin (type B, 150 Bloom) was obtained from Nutrition SC Co., Ltd. (Nakhon Pathom, Thailand). Dimethyl sulfoxide (DMSO) was purchased from RCI Labscan Limited, Bangkok, Thailand. Chitosan powder (80,000 MW), carboxymethyl cellulose (CMC), Tween-80 and plate count agar (PCA) were obtained from Sigma-Aldrich (St. Louis, MO, USA). Glycerol was purchased from Merck (Darmstadt, Germany). All the chemicals used in this study were of analytical grade.

### 2.2. Preparation of Red Cabbage Extract

Red cabbage samples without damage were brought to the Food Technology Laboratory at Mae Fah Luang University, Chiang Rai, Thailand, and washed with distilled water to remove dust and dirt. Red cabbage extract (RCE) was prepared according to Pereira et al. [17] with slight modifications. Red cabbage samples after size reduction were soaked in 70% ethanol with a sample to solvent ratio of 1:10 (*w*/*v*) and were kept overnight at 5 °C. The next day, ethanol was removed using a rotary evaporator, followed by freeze drying (Beta 2–8 LD plus, Martin Christ, Germany) for 48 h, and stored in a light-impermeable plastic bag at −20 °C until use for incorporation in intelligent film fabrication.

### 2.3. Preparation of Intelligent and Biodegradable Gelatin-, CMC-, Chitosan-Based Monolayer Films and Multi-Layer Films Fortified with RCE

Gelatin-, CMC- and chitosan-based monolayer films were developed and added with 0.5% (*w*/*v*) of RCE. Briefly, gelatin (2%, *w*/*v*), CMC (2%, *w*/*v*) and chitosan (2%, *w*/*v*) were added with glycerol 0.5% (*w*/*v*) in 100 mL for each biopolymer film. All the film-forming solutions (FFS) added with RCE 0.5% (*w*/*v*) were thermally dissolved, followed by pouring into molds and drying under fan for 24 h. Similarly, three layers were cast step-wise to develop a multi-layer film using gelatin, CMC and chitosan biopolymers. A porous gelatin-based layer as the innermost layer was prepared by using the micro bubble technique [18]. Gelatin (2%; *w*/*v*) was mixed with 100 mL of distilled water and 0.5% (*w*/*v*) glycerol. The mixture of gelatin FFS for the inner layer was dissolved at 80 °C for 15 min. The mixture was cooled before homogenization to generate bubbles at 20,000 rpm for 3 min. Gelatin FFS was then poured into the mold and kept overnight at 4 °C. The chitosan FFS for the middle or pH-sensitive layer incorporated with RCE was prepared by dissolving 0.5% (*w*/*v*) glycerol and 2% (*w*/*v*) chitosan in 100 mL of distilled water, and stirring was continued overnight and then added with 0.5% (*w*/*v*) of RCE before pouring it into the mold over the porous gelatin layer. Finally, it was left under a fan for 24 h before pouring the outer layer. The CMC-based FFS for the non-porous outer layer was produced by mixing 100 mL of distilled water with 0.5% (*w*/*v*) glycerol and 2% (*w*/*v*) CMC and dissolved at 60 °C for 20 min. The CMC-based FFS of the non-porous outer layer was cast on pre-casted layers of the same mold and allowed to dry under a fan for 24 h. All the monolayer and multi-layer film samples were finally conditioned in a humidifier at relative humidity (RH) of 45% with a humidifier temperature of 25 °C [19].

### 2.4. Characterization of Intelligent and Biodegradable Film

#### 2.4.1. Film Thickness and Microstructure

Film thickness was measured by a micrometer (Bial Pipe Gauge, Peacock Co., Tokyo, Japan) at nine random positions on the film as described by Muhammad et al. [20]. The microstructural morphology of the surface and the cross-section of pH-sensitive monolayer and multi-layered film samples were evaluated using a scanning electron microscope (Jeol, JSM-6610LV, Peabody, MA, USA), under an accelerating voltage of 10 kV at 500× magnification [21].

#### 2.4.2. Mechanical Properties

The tensile strength (TS) and elongation at break (EAB) of films were determined by a Universal Testing Machine (Lloyd Instrument, Hampshire, UK). Before testing, each film sample was cut into sizes of 20 × 50 mm^2^ and conditioned at 50% RH at 25 °C for 48 h. The test was carried out using a cross-head speed of 30 mm/min, a 1 kN load cell and an initial grip length of 30 mm (ASTM D882, 2012).

#### 2.4.3. Water Vapor Permeability (WVP)

The WVP of the films was measured by using a modified ASTM method (1989) as described by Kaewprachu et al. [22]. All the film samples were sealed in a permeation cup filled with dried silica gel (0% RH). The cups were placed in a desiccator saturated with water vapor at 30 °C and then weighed at 1 h intervals over a time period of 8 h. WVP was calculated using Equation (1):

WVP = WXA^−1^ t^−1^(P2 − P1)^−1^ (1)

W is the weight gain of the cup (g); X is the film thickness (mm); A is the area of exposed film (cm^2^); t is the time of film exposure (h); (P2 − P1)^−1^ is the vapor pressure differential across the film (Pa). The WVP is expressed as g mm h^−1^ cm^−2^ Pa^−1^.

#### 2.4.4. Moisture Content

The moisture content of the monolayer and multi-layered film samples was analyzed following the method of Zhang et al. [23]. The film samples were cut into 20 × 20 mm^2^ and weighed before drying in a hot air oven at 105 °C for 24 h.

#### 2.4.5. Film Solubility

The film solubility was determined according to the method of Kaewprachu et al. [22] with slight modifications. The conditioned monolayer and multi-layered film samples of 20 × 20 mm^2^ were weighed and placed in a 50 mL centrifuge tube containing 10 mL of distilled water. The mixture was shaken at a speed of 250 rpm using a shaker (HeidolthInkubator 10000, Schwabach, Germany) for 24 h. The un-dissolved debris were then removed by centrifugation at 3000× *g* for 30 min. The residual film samples were dried in a hot air oven set at 105 °C for 24 h and weighed to determine the solubility of the film by Equation (2):

Film solubility = 100 × (W_i_ − W_f_)/W_i_ (2)

where W_i_ is the initial weight of the film and W_f_ is the final weight of the film after drying.

#### 2.4.6. Color Properties, Light Transmission and Film Transparency

The color values of the film were determined using Color Quest XE (Hunter Lab, Reston, VA, USA). The values of L* (lightness), a* (red-green) and b* (yellow-blue) were recorded. The UV light barrier properties of the films were measured [22] using a UV-16001 spectrophotometer (G105 UV-VIS, Thermo Scientific Inc., Waltham, NJ, USA) at a wavelength between 200 nm and 800 nm, and the transparency of the film was calculated using Equation (3):

Transparency = −log T_600_/X (3)

where T_600_ = absorbance at 600 nm; X = average thickness of the film.

#### 2.4.7. PH Sensitivity and Gas Sensitivity of the Film

The pH sensitivity of the film was determined according to the method described by Pereira Jr, de Arruda and Stefani [18] with some modifications. First, a 2 × 2 cm^2^ film was placed in a small Petri dish to be submerged in pH buffer solution (pH 1 to 12) prepared using 0.1 M and 0.2 M hydrochloric acid, 0.1 M and 0.2 M sodium hydroxide, 0.2 M potassium chloride, 0.1 M acetic acid, 0.1 M sodium acetate, and 0.1 M disodium hydrogen phosphate. After 10 min, photographs of the film samples were recorded. NH_3_ was used to analyze gas sensitivity based on the color response of the chitosan and multi-layer film samples to basic gas. Briefly, the films (10 × 10 mm^2^) were placed in an Erlenmeyer flask (500 mL) containing ammonia solution for 30 min at 25 °C. The photos of the films were captured using an optical scanner (Scanjet G4050, HP, Palo Alto, CA, USA) and the stability of the films was determined with the color change [24].

#### 2.4.8. Dye Leaching Test and Biodegradability Test

Dye leaching from the film samples was evaluated as per the method of Lee et al. [25]. According to Regulation (EU) No. 10/2011 related to the migration testing of packaging material in contact with food, the food simulants for fresh and refrigerated fish are water and 95% ethanol. Thus, the monolayer and multi-layer film samples were soaked in 50 mL beakers according to the food simulants used, at 60 °C for 10 days. The dried film and the solution were then analyzed using Fourier transform infrared spectroscopy (FT-IR, Spectrum 65 FT-IR Spectrometer, Perkin Elmer Co., Akron, OH, USA) to detect the migration of anthocyanins onto the food surface. The biodegradability of the monolayer and multi-layered film samples was determined by placing pieces of 40 × 40 mm^2^ film samples in glass jars containing soil compost. Film samples were incubated under a controlled temperature and relative humidity of 58 ± 2 °C and 50 ± 10% RH [26] for a period of 30 days. After the biodegradation time period, the disintegrated samples were observed under an optical microscope.

### 2.5. Application of Intelligent and Biodegradable Multi-Layer Film on the Freshness of Tilapia Fish Fillets

#### 2.5.1. Fish Spoilage Trial

To perform the application test on fish, the developed multi-layered film (40 × 40 mm^2^) was fixed on the headspace of sterile Petri plates containing 25 g of fish fillet samples. The plates were then closed tightly with paraffin film and placed in an incubator at 4 °C for a period of 10 days. Chemical and microbial analyses were every two days of storage.

#### 2.5.2. Total Volatile Basic Nitrogen (TVB-N) Content

The TVB-N level of the fish samples was measured by the Kjeldahl method [27]. Briefly, 10 g of fish was blended with 10 mL of water and the mixture was homogenized and filtered using filter paper (Whatman no. 1). Then, 5 mL of magnesium oxide suspension (10 g/L) was added into the reaction chamber with 5 mL of the filtrate. In the blank experiment, distilled water was used to replace the sample. The condensate tube was inserted into 100 mL of 2% boric acid solution. After the reaction was terminated, the boric acid solution was titrated with 0.01 M hydrochloric acid solution until the color became blue-violet. The TVB-N content was then calculated.

#### 2.5.3. Microbial Analysis

Total viable count (TVC) was analyzed to indicate the microbial quality and freshness of fish samples [27]. Every 2 days of storage, a 10 g sample was aseptically transferred to a sterile bag and added with 90 mL of sterile 0.1% bacteriological peptone water. The mixture was homogenized in a stomacher for 2 min at room temperature. Serial dilutions using 0.1% sterile peptone water were prepared in triplicates and 1 mL of each sample at an appropriate dilution factor was poured onto bacteriological agar plates. Incubation was carried out at 37 °C for 48 h, and the microbial load is reported as CFU/g of sample.

### 2.6. Statistical Analysis

Statistical analysis was performed using SPSS for Windows (SPSS Inc., Chicago, IL, USA) to test the analysis of variance (ANOVA). At a 95% confidence level, Duncan’s multiple range tests were used to identify the significant difference between treatments. Statistical analysis was performed for physical properties (color, transparency, moisture content, water vapor permeability) and mechanical properties (thickness, tensile strength, elongation at break); the solubility of the films was also analyzed statistically as a study variable.

## 3. Results and Discussion

### 3.1. Thickness and Microstructure of Gelatin-, CMC-, Chitosan-Based Monolayer Films and Multi-Layer Films Fortified with RCE

The thickness of the monolayer films prepared from gelatin, CMC and chitosan ranged from 0.034 mm to 0.040 mm (*p* > 0.05) (Table 1). The multi-layer film composed of gelatin, CMC and chitosan with 0.5% (*w*/*v*) of RCE showed the highest thickness of 0.123 mm compared to the monolayer films (*p* < 0.05). Film thickness could improve the light barrier, the water vapor transmission rate and mechanical properties. Chitosan film supplemented with 0.3% of black soybean seed coat extract exhibited a higher thickness than plain chitosan film [28]. Polyphenol hydroxyl groups and anthocyanins could function as a bridge and closely combine with chitosan chains through intermolecular interactions [29]. Thus, the RCE could be well distributed among chitosan molecules to impart higher thickness in the multi-layer film.

**Figure 1:**
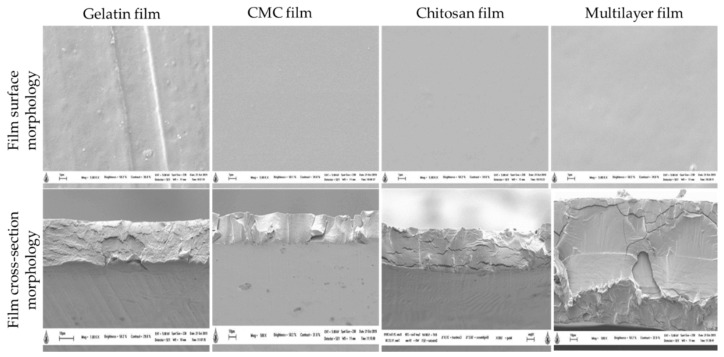
Microstructure of surface morphology and cross-section of the developed film at 500× magnification.

The microstructure of the film surface morphology observed under a scanning electron microscope presented smooth surfaces for CMC, chitosan and multi-layer films, indicating the high compatibility of molecular interactions between the materials (Figure 1). However, slight roughness in the film surface was observed in the gelatin-based monolayer film. The smoothness of the surface and cross-section in chitosan film was reported because of the high compatibility between chitosan matrix and glycerol [30]. In the case of cross-section morphology, microstructure images of monolayer films displayed thinner cross-sections than the multi-layered films. The thickness observed from the cross-section morphology was correlated with the highest thickness values obtained in multi-layered film in which three bio-polymers completely sandwiched the RCE-containing chitosan layer (Table 1). Notably, several spots were evenly distributed on the surfaces of chitosan incorporated with purple corn extract, and the visualized spots were attributed primarily to anthocyanin aggregation [28]. The multi-layer film had a smoother and thicker cross-section, possibly due to the hydrophilic property of anthocyanins in RCE, and it was more compatible with the chitosan matrix sandwiched between gelatin and CMC films. Chitosan has been reported to plasticize more efficiently with glycerol [31]. Additionally, the addition of RCE anthocyanin to the film base provided a higher chance of bond formation, particularly hydrogen bonding, thereby improving the surface of the polysaccharide matrix with a smooth appearance [23].

### 3.2. Mechanical and Physio-Chemical Properties of Gelatin-, CMC-, Chitosan-Based Monolayer Films and Multi-Layer Films Added with RCE

#### 3.2.1. Mechanical Properties

As for the mechanical properties of the film, the highest tensile strength of the monolayer chitosan incorporated with anthocyanin was 21.76 MPa (Table 1). According to Zhang, Zou, Zhai, Huang, Jiang and Holmes [23], the addition of anthocyanin to the chitosan base allows the formation of more hydrogen bonding between the hydroxyl group of the anthocyanin and the amino group of the chitosan, thereby improving the tensile strength of the film. Simultaneously, the addition of anthocyanin further reduces the water interaction within the chitosan matrix, due to which there was a decrease in the value of elongation at break (Table 1). For the multi-layered film, the tensile strength decreased when compared to the monolayer chitosan film. A study revealed that the increase in the molecular weight of a film will decrease the tensile strength of the film and thereby simultaneously increase the elongation at break property of the film [32]. Moreover, the increased tensile strength in chitosan film added with 0.5% of RCE was related with the compact and dense cross-sectional morphology of chitosan and the multi-layer film, shown in Figure 1. The addition of 1% (*w*/*w*) of Chinese bayberry (*Myrica rubra* Sieb. et Zucc.) into the cassava film matrix resulted in a dense and compact internal microstructure, which greatly improved the water vapor permeability and tensile strength of the film [10].

#### 3.2.2. Physical Properties of Intelligent Films

Regarding the water vapor permeability (WVP) of the film, it was noticed that the multi-layered film showed the highest response when compared to the monolayer film samples (Table 1). According to Yong et al. [33], WVP is a vital barrier parameter reflecting the ability of the film against the transmission of water vapor. The multi-layer film formation includes the usage of a porous inner layer to allow the permeation of the metabolites and water vapor, thereby showing the highest WVP values. In terms of the moisture content of the film, the monolayer chitosan film incorporated with RCE showed the highest moisture content value of 24.41%. The increase in the moisture content of the film is due to the hydrophilic nature of the anthocyanin [23]. The availability of more polar sites provides more opportunities to absorb moisture in a monolayer chitosan film that showed the highest moisture content value [30]. In the case of the multi-layered film, a decrease in the moisture content was noticed compared to the monolayer film. Despite the anthocyanins being added to the dye-incorporated layer of the multi-layer film, the polar sites of the anthocyanins were not able to absorb moisture sufficiently from the atmosphere due to the outer layer made of CMC, which prevented the absorption of moisture from the environment. Qin, Liu, Yuan, Yong and Liu [31] reported that the reduction in the moisture content of the film could be due to the interaction between the high abundance of hydroxyl groups of the anthocyanins and the hydroxyl and amino groups of the chitosan, reducing the possibility of moisture absorption based on the polar active sites, unlike in the monolayer film. The solubility of the film samples was analyzed, as shown in Table 1. Bio-based films need to be durable for packaging with minimum solubility [34]. Solubility gives an indication of the film’s water affinity [35]. The solubility of the multi-layer film was the lowest in comparison to the monolayer films made from gelatin, chitosan and CMC materials added with RCE.

The lowest transmittance and transparency values were obtained in multi-layer film, indicating the partial opacity of three distinct layers, as shown in Table 2. The light transparency reflects the barrier property of a film against UV–vis light that could be detrimental for food storage [36]. The film transparency and transmittance are important factors to determine the light barrier properties affecting a product’s appearance when used as a packaging material. A decrease in transparency was observed after increasing the amount of sappan (*Caesalpinia sappan* L.) heartwood extract incorporated in gelatin film [37]. The decrease in transparency was reported with the decrease in lightness of active chitosan/PVA films with anthocyanins from red cabbage (*Brassica oleraceae*) as time–temperature indicators for application in intelligent food packaging [17]. Kaewprachu et al. [38] reported similar findings of catechin–Kradon extract addition, which decreased the transparency of fish myofibrillar protein films. Appearances and colors of the film samples are presented in Table 3. Color is another important parameter in the development of the intelligent freshness indicator films since the concept of the indicator is based on the color-changing system of the film. The CIE L* a* b* color model was selected for measurements of the film color. According to the concept of CIE, the L* value is an indicator of lightness if high, or darkness if low. The a* value represents redness when positive, and greenness when negative. The b* value is an indicator of yellowness when positive, and blueness when negative. No difference was observed visually between the monolayer chitosan film and the multi-layer film.

#### 3.2.3. Chemical Characteristics of Intelligent Films

**Figure 2:**
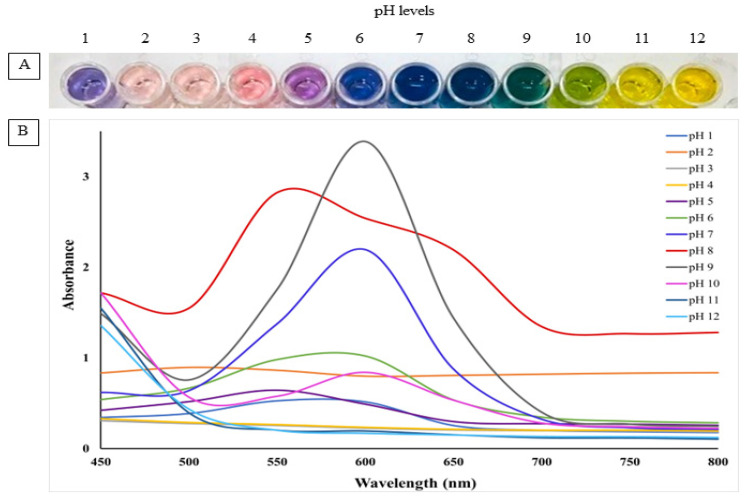
pH sensitivity (**A**) and UV–Vis (G105 UV-VIS, Thermo Scientific Inc., Waltham, NJ, USA) absorption spectra (**B**) of the red cabbage extract at 1–12 levels of pH.

**Figure 3:**
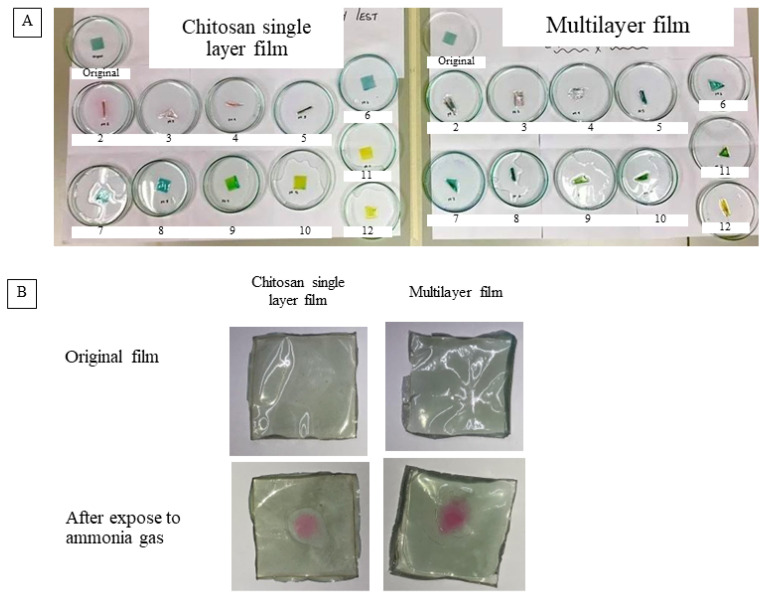
pH sensitivity ((**A**) numbers in each figure are pH value) and gas sensitivity (**B**) of the monolayer chitosan film vs. multi-layered films.

RCE powder was tested for pH sensitivity, as shown in Figure 2A and Figure 3A. The color changes in different pH conditions, turning from deep orange red to light carmine over the pH range of 2–7, and then purple at pH 8–9, yellow-green at pH 10 and finally yellow at pH 11–12. Corresponding to color changes in RCE solutions, the maximum absorption peak exhibited red shifts along with the increases in pH. The change in the color of the anthocyanin could be associated with the change in the structure of functional groups in RCE upon being exposed to different pH values. pH sensitivity results of RCE were evaluated in the form of absorption spectra, as shown in Figure 2B. The maximum or the highest absorption peak was obtained between 500 and 600 nm. The specific peak at 600 nm indicated an alkaline environment. Anthocyanins changed color based on the changes in pH of the medium [39]. Generally, anthocyanins show flavylium cation (red, at strong acidic condition), carbonil psuedobase (colorless, at weak acidic conditions), quinodal base (colorless, at weak alkaline conditions) and chalcone (yellow, at strong alkaline conditions) [35]. On the other hand, the monolayer and multi-layered films showed similar color changes when the films were soaked in different pH buffer solutions. At a lower pH value, the films showed a pink color, indicating the presence of flavylium cation structure. With the increase in the pH value, the color changed to green (quinodal base) and then finally to yellow (chalcone state). Although the multi-layered film is a combination of three distinct layers, the pH sensitivity test result showed similar trends to that of the monolayer chitosan film incorporated with anthocyanins. The composite film fabricated from gelatin, gellan gum and red radish anthocyanin extracts showed similar pH-sensitive color changes [3].

Gas sensitivity tests of the chitosan film and multi-layer film were analyzed as shown in Figure 3B. The ammonia gas generated in the test tube using ammonium chloride and calcium hydroxide was entrapped by the film at the top of the test tube. Both the monolayer chitosan film incorporated with RCE and the multi-layer film responded positively by changing color from green to pink when exposed to the ammonia gas. Intelligent film produced from whole arrowroot powder, soy protein isolate and food-grade red cabbage anthocyanins showed distinguishable color changes in different pH buffers and good sensitivity for ammonia vapor detection [40].

**Figure 4:**
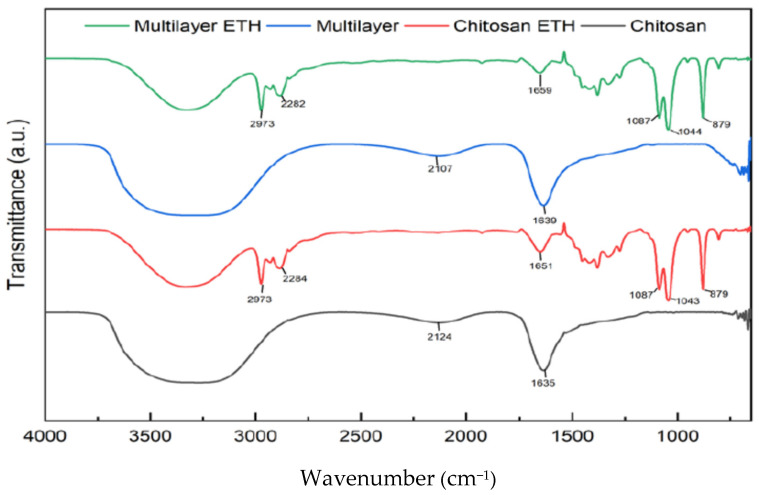
FTIR spectra of chitosan film and multi-layered film soaked in water and FTIR spectra of chitosan film and multi-layered film soaked in ethanol.

Dye leaching is another important parameter that was analyzed in monolayer and multi-layered intelligent films. According to the Food and Drug Administration, the most common food stimulants for fresh and refrigerated fish are water and ethanol. Chitosan and multi-layer film samples were soaked in the respective aqueous and ethanol solvents for 10 days. The leaching of RCE dye normally occurs in monolayer film samples. So, this test was carried out to verify and assure the safe use of the multi-layer intelligent film for food applications. Then, the FTIR analysis for both the dried films and the solution samples was performed. FTIR spectra were analyzed to verify or spot the presence of the functional group of the anthocyanins in both the dried film and the solution sample in which the film was soaked for 10 days. The FTIR analysis for the film was to ensure that anthocyanins from RCE were retained in both the monolayer and the multi-layer film. Similarly, FTIR analysis was performed for the residual solution to verify the leached anthocyanins into the solution when exposed to different food stimulants. Figure 4 shows the FTIR spectra of the RCE anthocyanin functional groups both from the dried film samples and the multi-layer sample. FTIR was employed to detect the anthocyanin functional groups in ethanolic plum extracts [41]. The film samples were incorporated with anthocyanin extract from RCE, and the dye leached in residual solutions could have similar functional groups, as shown in Figure 4. When the monolayer chitosan film was soaked in ethanol and water solutions for 10 days, the FTIR result of the monolayer film in both the stimulants showed that anthocyanins were leached out during the soaking process. As can be observed from Figure 4, the solution sample of chitosan film in water showed the presence of two distinct peaks at 2877.14 cm^−1^ and 3267.01 cm^−1^, indicating the stretching vibration between N-H and O-H bonds due to the interaction between the chitosan and the anthocyanin. When the same chitosan film was soaked in the ethanol solution, shifts in the chitosan peaks were observed at values from 3291.31 cm^−1^ and 1027.05 cm^−1^ to 3330.98 cm^−1^ and 1053 cm^−1^, respectively. The presence of the peaks at 3418 cm^−1^, 1639 cm^−1^ and 1053 cm^−1^ showed the possibility of the presence of the functional groups of anthocyanins [42]. Comparing Figure 4, the FTIR analysis of the solution samples of the monolayer chitosan film showed similar peaks, so we can conclude that the anthocyanin dye incorporated into the monolayer film was leached out in both types of stimulants. FTIR spectra were evaluated in the gelatin-based intelligent film in which the addition of sappan (*Caesalpinia sappan* L.) heartwood extract can form a hydrogen bond with a related functional group to reduce free hydrogen that can form hydrophilic bonds with water [39].

Similarly, the multi-layer film was also soaked in both water and ethanol as stimulants. Using water as the solvent, even the multi-layer film showed positive test results. As can be seen from Figure 4, when the dried sample was analyzed, the highest peak obtained was at 1022.01 cm^−1^. However, after the sample solution was analyzed, new peaks were observed at 3266.94 cm^−1^ and 1634.70 cm^−1^, indicating shifts caused in the peaks due to the interactions between the anthocyanins and the polymer matrices, thereby indicating that the dye was leached from the multi-layer film as well when water was used as a stimulant. Moreover, when the multi-layered film was soaked in ethanol and water, the film solution of ethanol gave a negative result, indicating that no dye was leached out despite the multi-layer films being soaked in ethanol solution for 10 days. As shown in Figure 4, when the dried film sample in ethanol was analyzed, three distinct peaks were observed at 3326.84 cm^−1^, 1044.02 cm^−1^ and 1380.26 cm^−1^. When the solution sample was analyzed, no specific peaks were obtained and the graph showed a stable line, indicating the absence of anthocyanins being leached from the multi-layer film. pH-sensitive polymeric dyes were fabricated by grafting covalently phenol red and rosolic acid onto the chitosan matrix and showed excellent color stability and dye leaching resistance [43].

**Figure 5:**
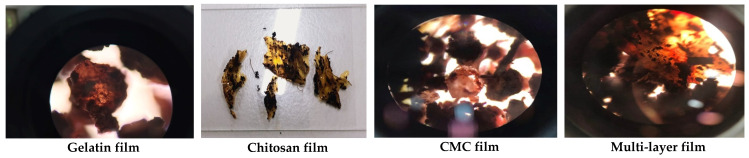
Photographs of disintegrated monolayer gelatin, chitosan and CMC films and multi-layered film observed under an optical microscope.

Biodegradability was confirmed by the disintegration of gelatin, CMC, chitosan monolayer and multi-layer intelligent films visualized under an optical microscope, as shown in Figure 5. After burying the four different types of films in the compost soil, samples were conditioned at 58 ± 2 °C, 50 ± 10% RH for a period of 30 days. All the film samples—gelatin-, chitosan- and CMC-based monolayer films and the multi-layered film—showed a positive response by degrading the films into smaller parts with complete deformation. From the optical microscopic (OM) images, gelatin, chitosan and CMC monolayer films showed similar patterns of disintegrated and deformed flakes after 30 days of composting time. However, the porous structure of morphology was visualized with intact deformation and slightly bigger flakes in the multi-layer film under OM. This could be related to the multi-layer arrangement and exposure to moisture adherence of different biopolymer layers in the multi-layer film sample to resist complete disintegration compared to the monolayer films. Additionally, gelatin, CMC and chitosan are bio-based hydrocolloids that have no negative impact on the environment and could be used as potential base materials for the development of intelligent packaging indicators to evaluate the freshness of perishable food. The biodegradability of gelatin/CMC/chitosan composite films was evaluated for 5 days in soil compost, which a confirmed significant loss of weight due to the deterioration of the film structure during the disintegration time [44]. A biodegradability test of the biopolymer film made of cassava and CMC incorporated with turmeric oil (30 μL) coated on a kraft paper reported faster degradation. The mechanism of rapid disintegration of the cassava/CMC composite containing turmeric oil could adhere polar groups of both the biopolymer and the essential oil with the water to form hydrogen bonds, thus accelerating the disintegration of the samples [45]. Similarly, the entry of soil microorganisms into the film was attributed to the increase in water sorption in the starch-based composite films [46].

### 3.3. Application of Intelligent Film on the Freshness and Microbial Quality of Tilapia Fish Fillets during 10 Days of Storage

**Figure 6:**
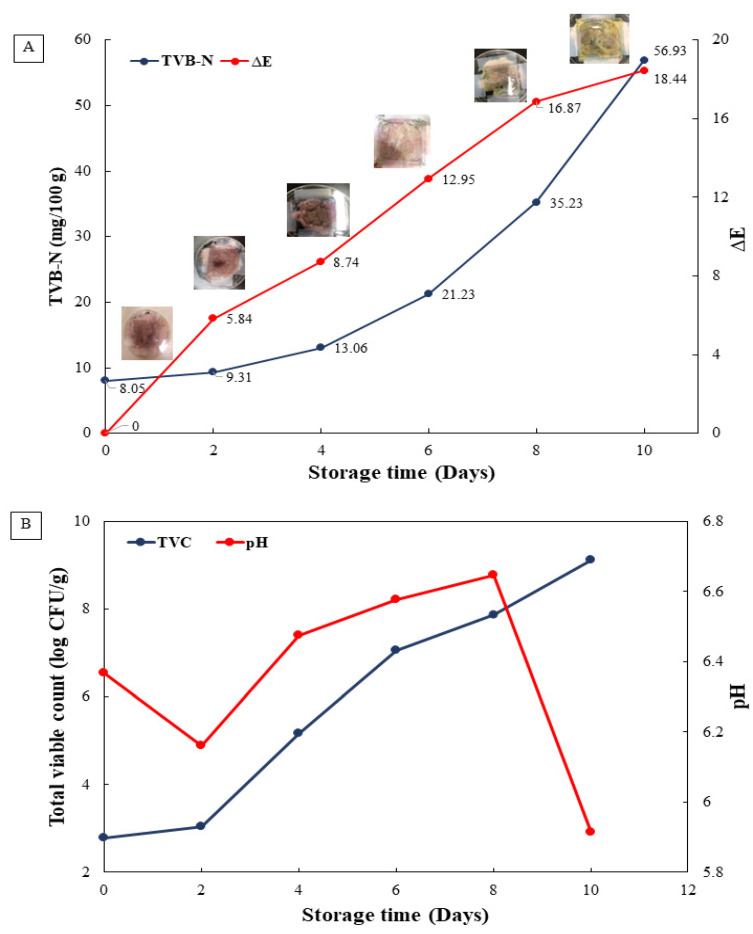
TVB-N content, color difference (del E) (**A**) and total viable count, pH (**B**) of the fish samples using intelligent multi-layer film for 10 days of storage at 4 °C.

The freshness and microbial quality of Nile tilapia fillets were monitored by using intelligent film samples incorporated with RCE and stored at 4 °C for 10 days (Figure 6A). The color of the multi-layer film changed possibly due to changes in the pH and the amine content of the sample (Figure 6B). Initially, the color of the film sample on day 0 was pink. With the duration of storage, fish fillets experienced spoilage and degradation of muscle proteins due to endogenous and microbial proteases producing volatile basic non-protein nitrogen compounds such as trimethyl amine (TMA).

The production of basic volatile nitrogen in the form of ammonia gas has been reported in degraded silver carp fish muscles during storage [47]. The multi-layer film sample detected the ammonia gas present in the packaging as the nitrogen content increased, changing the color of the films from pink on day 0 to blue on day 6, green on day 8 and finally yellow on day 10. The color changes during storage intervals indicated that the samples degraded due to various external factors such as microbial spoilage and proteolytic enzyme-assisted reactions, further contributing to autolysis of the fish muscles [48,49].

TVB-N value is widely used as a quality parameter to evaluate the freshness of fish and meat products [50]. The average rejection limit of TVB-N for fish ranges between 20 and 30 mg/100 g depending on the composition of samples [51]. This further instigates that the rejection level in its lower limit for TVB-N in fishes is 20 mg/100 g. As shown in Figure 6A, TVB-N content increased from 8.05 mg/100 g on day 0 to 13.06 mg/100 g on day 4 of refrigerated storage; the sample experienced slow degradation with lower microbial load and was approved for safe consumption. However, after 6 to 10 days of storage, TVB-N amount exceeded the rejection limit, from 21.23 mg/100 g to 56.39 mg/100 g. The multi-layer intelligent film color was in accordance with the results of the pH sensitivity test in which a yellow color was attained in high pH conditions. Thus, TVB-N content was correlated with a change in pH and the degradation of fish muscles releasing basic nitrogen volatile compounds to be sensed by the color differences in RCE in the intelligent multi-layer film during 10 days of storage. The trimethylamine (TMA), dimethylamine (DMA) and ammonia (NH_3_), collectively known as total volatile basic nitrogen, were detected using plastic film incorporated with bromophenol dye as a pH-sensing indicator of fish freshness, placed in the head space of the package, showing a gradual color change and increased bacterial load during fish spoilage [52].

Figure 6B presents the average number of microbes that increased dramatically from day 0 to day 10 of refrigerated storage in tilapia fish fillets. The efficacy of the modified atmosphere composed of argon, carbon dioxide and oxygen along with non-thermal cold plasma on the microbial load retarded the microbial load for up to 6 days in tilapia fish fillets during refrigerated storage [53]. The average number of colonies for microbial spoilage in fish should be less than 5 log CFU/g, according to the fish quality control department of Thailand. On day 4, the microbial count already exceeded the specified limit, indicating that fish spoilage had begun. As the fish spoils, the organic compounds in it degrade, allowing microbes to extract nutrients and thrive in that environment. This further degrades the protein into different nitrogenous compounds, causing the production of volatile gases for detection. In addition to pH sensitivity of tilapia fish fillets, other causes of tilapia spoilage such as protein degradation or microbial spoilage, allowing the production of both acidic and basic by-products, result in incremented pH of the tilapia sample. The increase in the pH is related to the formation of basic compounds such as ammonia and trimethylamine (TMA), induced by microbial proliferation in tilapia during storage [53].

## 4. Conclusions

We developed biopolymer-based intelligent monolayer and multi-layered films incorporated with RCE to act as a natural pH-sensing indicator for food applications. Gelatin-, chitosan- and CMC-based monolayer films and a multi-layered intelligent film were tested for their physico-chemical, microstructural and mechanical properties. The multi-layered film yielded the highest results compared to gelatin-, CMC- and chitosan-based monolayer films. The microstructure analysis revealed thicker cross-sections in the multi-layer film than the other films. Chitosan film has higher tensile strength than the multi-layer film, CMC and gelatin films. Elongation at break was slightly higher in CMC compared to the multi-layer film and gelatin film. WVP was higher in the multi-layer film than the other monolayer films. Moisture content was highest in chitosan film, followed by the multi-layered film and other monolayer films. CMC film showed the highest solubility compared to multi-layered and chitosan films. Additionally, transmittance and transparency values in the multi-layered film were the lowest compared to the chitosan-, CMC- and gelatin-based films. L* and a* values decreased, while b* values increased in the multi-layered film compared to the other film samples. pH sensitivity and ammonia gas tests showed no changes in the chitosan and multi-layer films. FTIR spectra confirmed that dye leaching was not detected in the ethanol-soaked multi-layered film. Biodegradability tests showed rapid degradation of multi-layered and chitosan films within 1 month. Therefore, the multi-layered film was employed to monitor the fresh quality of tilapia fish fillets at 4 °C for 10 days. The results of freshness acceptability were noted on day 6 due to a change in the color of the multi-layered film and as evidenced by higher TVB-N content of 21.23 mg/100 g in stored tilapia fish fillets. Thus, the multi-layered film could be used as a natural and safe indicator to sense the quality of the fish freshness without leaching dye onto the fish muscle.

## Figures and Tables

**Table 1:** Mechanical and physico-chemical properties of the biopolymer-based monolayer and multi-layer films incorporated with RCE.

Film Samples	Thickness (mm)	Tensile Strength (MPa)	Elongation at Break (%)	WVP (10^−5^/10^−6^ g mm h^−1^ cm^−2^ P^−1^)	Moisture Content (%)	Solubility (%)
Gelatin	0.035 ± 0.01 ^b^	9.15 ± 1.40 ^d^	9.61 ± 11.1 ^c^	1.84 ± 0.56	16.68 ± 1.52 ^c^	37.61 ± 1.03 ^b^
CMC	0.040 ± 0.01 ^b^	15.89 ± 1.50 ^c^	35.67 ± 7.62 ^a^	5.15 ± 0.79	19.54 ± 1.12 ^b c^	77.41 ± 0.94 ^a^
Chitosan	0.034 ± 0.01 ^b^	21.76 ± 2.71 ^a^	23.54 ± 5.15 ^b^	5.56 ± 1.20	24.41 ± 2.36 ^a^	24.94 ± 4.11 ^c^
Multi-layered	0.123 ± 0.00 ^a^	18.73 ± 2.71 ^b^	33.12 ± 9.88 ^a^	1.24 ± 0.05	20.12 ± 0.59 ^b^	23.19 ± 1.13 ^c^

Values are expressed as means ± SD (*n* = 3). Different letters in the same column indicate significant difference at *p* < 0.05.

**Table 2:** Light transmission and transparency of the biopolymer-based monolayer and multi-layer films incorporated with RCE.

Film Base	Transmittance at Different Wavelengths (nm)				Transparency%
	200 nm	400 nm	600 nm	800 nm	
Gelatin	58.93	66.88	75.03	81.10	7.91 ± 0.04 ^a^
CMC	48.60	56.41	65.77	69.83	3.34 ± 0.09 ^c^
Chitosan	37.67	44.76	62.40	76.46	6.01 ± 0.19 ^b^
Multi-layered	27.60	37.50	47.90	66.80	1.32 ± 0.08 ^d^

Values are expressed as means ± SD (n = 3). Different letters in the last column indicate significant difference at *p* < 0.05.

**Table 3:** The appearance and color attributes of the monolayer and multi-layer films incorporated with RCE.

Film Samples	Appearance	L*	a*	b*
Gelatin	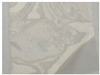	95.02 ± 0.62 ^a^	−1.60 ± 0.10 ^a^	2.09 ± 0.34 ^b^
CMC	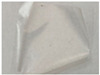	98.58 ± 0.38 ^a^	−1.35 ± 0.01 ^a^	0.97 ± 0.02 ^c^
Chitosan	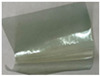	89.34 ± 1.09 ^b^	−12.40 ± 0.89 ^b^	3.91 ± 0.27 ^a^
Multilayer	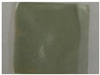	85.26 ± 1.33 ^c^	−13.77 ± 0.74 ^c^	4.11 ± 0.57 ^a^

Values are expressed as means ± SD (n = 3). Different letters in the same column indicate significant difference at *p* < 0.005.

## Data Availability

The data presented in this study are available on request from the corresponding author.
